# Changes in food purchase, consumption and handling during COVID-19 pandemic among single person households

**DOI:** 10.1371/journal.pone.0294361

**Published:** 2023-11-29

**Authors:** Janet Antwi, Yetunde Olawuyi, Shadiamon Bain, Kyndall Samuel

**Affiliations:** Department of Agriculture, Nutrition and Human Ecology, Prairie View A&M University, Prairie View, TX, United States of America; Wroclaw University of Environmental and Life Sciences: Uniwersytet Przyrodniczy we Wroclawiu, POLAND

## Abstract

**Objective:**

As a new type of consumer subject in the market that was formerly dominated by multiple person families, single households are driving the change in the buying structure. Food purchase activities have undergone significant changes since the outbreak of the COVID‐19. The objective of this study was to assess and compare variations in food consumption, purchase and handling during the COVID-19 pandemic between single person households (SPH) and multiple person households.

**Method:**

A cross-sectional study conducted among 211 individuals in communities in Harris and Waller Counties, Texas. Sociodemographic, food purchase, food consumption and food handling activities during the COVID-19 pandemic were assessed with a validated COVID-19 Nutrition questionnaire.

**Results:**

Non-Hispanic Black participants constituted 42.6%, and 28.4% were Hispanics. Participants were made up of mostly aged 18–24 years (39.3%), 47.9% single household composition, 30.4% in full time employment, and 29.1% partook in food assistance programs. A large proportion of them had never used grocery pickup services, online grocery shopping and a farmers’ market. During the COVID-19 pandemic, majority of the participants used more of large supermarkets, restaurant/fast food, and online grocery but food consumption seemed to remain the same for the majority of participants. For beverages, majority of participants consumed more water, less soda, and no alcohol. There was a significant association between single person household and higher restaurant/fast foods purchase. Many of the participants reported weight gain and less physical activity during the pandemic.

**Conclusion:**

Restaurant meal purchases was more prevalent in single-person families. The results from the study have the potential to contribute to how public policy officials, food service, and health authorities forecast how different categories of consumers will react in pandemics and may be used to inform area-specific alleviation strategies to minimize the impact of the COVID-19 pandemic and future events.

## Introduction

The single person household (SPH), which refers to a household that contains one person who lives alone has become a growing demographic in the US [1]. This trend is not only seen in the US, single-person households have become increasingly common in many countries across the world [2]. According to a report [3], 37 million (15%) adults aged 18 and above lived alone in early 2021 in the US, a rise from 33 million (14%) in 2011. This represents 28% of all U.S. households in 2021, whereas only 13% of all US households in 1960 were SPHs. This growing trend has been attributed to changes in demographic behaviors, institutional arrangements, and labor migration in the past few decades [4]. Other hypothesized economic and sociocultural variables influencing the rise in SPHs include individualism, late marriages, low birth rates, and an elderly population, as well as employment opportunities for women [5,6] this makes the SPH a very diverse group [7]. Over the period of 2016–2030, there will be an estimated 120 million additional SPHs, representing a greater rate of increase than any other type of household in the world [8]. The rise in SPH has raised serious concerns among policymakers and academics due to the significant implications for a society’s institutional framework and its citizens’ well-being, including the effectiveness of a society’s resource distribution or welfare system, the operation of the family system, and its citizens’ physical and psychological well-being [6]. Since the fastest-growing household group now includes individuals, or SPHs, it has been hypothesized that shifting household demographics are the main cause of the unexpected rise in popularity of meal delivery apps [9].

SPHs have a unique food-related consuming behavior; they are consumers who develop an original consumer culture. Newly coined words referring to them have emerged, such as “FIT” (free intelligent tribe) [9]. Previously dominated by families with multiple people, single person households are driving the shift in the consumption structure as a new type of consuming subject in the market [10]. There is data to suggest that single person and multiple person families differ significantly in terms of their shopping attitudes and practices [5]. SPHs exhibits features of generous spending because, in comparison to other household types, they have adequate time, space, and money to spend [11]. Previous research have demonstrated that they typically like eating out or consuming purchased meals over preparing their own meals [8,12,13]. Age, education, household composition, and income have all been found to have a significant impact on fast food consumption. A single person home had a 1.4 percent higher chance of purchasing fast food on a particular day in 2003–2011 compared to married and unmarried couple households without children [13]. SPH have been divided into three categories depending on their eating habits: those who prioritize convenience, those who prioritize their well-being when dining out, and those who don’t participate [6,14]. SPHs are the primary consumers of food prepared away from home [15]. Opportunities exist for the foodservice industry and food producers, as the proportion of households with just one person rises. SPHs have a significant need for single-portion, portable, frozen or ready meals. Wealthy singles are more likely to frequent restaurants and are prepared to pay more for high-quality cuisine [16].

Since the coronavirus (COVID-19) epidemic in early 2020 [17], consumer food buying habits have undergone substantial modifications. According to earlier studies, the COVID-19 pandemic had an impact on how Americans bought food. American households reportedly stopped eating at full-service restaurants and instead bought more food and drinks at supermarkets and other places where food is prepared at home (FAH) [18]. Numerous studies on consumers’ eating and shopping habits during the pandemic consistently demonstrate that consumers’ increased spending at grocery stores, supermarkets, and other food-at-home (FAH) establishments only partially offset their lower spending at restaurants and other food-away-from-home (FAFH) establishments [15]. By the end of March 2020, new restrictions rendered physical store shopping disadvantageous. In-store shopping was not only viewed as high risk, but it was also tough to visit a single store and find it fully supplied due to additional restrictions imposed by businesses or municipal legislation. As a result, customers began utilizing internet services. Distribution facilities became overburdened due to the spike in demand for online food transactions. Food shopping looked vastly different before the COVID‐19 confinements in contrast to post COVID‐19 confinements, and a study reported a decrease in the number of processed foods consumed post COVID‐19 confinements [19]. Studies based on surveys of American households or adults also support the finding that eating out results in lower spending and food consumption. Some people discovered that this decline only applied to fast foods in particular [15,20–22].

Diverging observations have been made in different populations, with some people making improvements with their diet quality such as reduction in consumption of red meat, processed food together with the increase of fruits and vegetables [23–25]. These observations demonstrate that the pandemic had different impacts on people’s lifestyle and food consumption patterns. Few studies have focused on the food behavior changes of SPH during the COVID-19 pandemic. We quantified the effect of the COVID-19 pandemic on the food consumption and handling practices of SPH to determine whether the lifestyle habits of our findings will differ from other populations and communities explored on the topic in the literature. The objective of this study was to assess and compare variations in food consumption, purchase and handling during the COVID-19 pandemic between single person households (SPH) and multiple person households.

## Materials and methods

### Study design and study area

We employed a cross-sectional study to assess changes in food consumption, purchase and handling during the COVID-19 pandemic. We collected data from participants who were residents in the Waller and Harris Counties in Houston, TX. The study protocol was approved by the Prairie View A&M University Institutional Review Board with IRB #2121–093. All participants were required to complete a written informed consent form prior to participation in the study.

### Study population and sampling

Two hundred and eleven (n = 211) individuals were recruited through convenient sampling from the study area to participate in the survey. The survey was conducted from January 2022, after the lifting of the COVID-19 related lockdown restrictions. The inclusion criteria were age of ≥18 years, and experience with changes in food purchasing and consumption during the COVID-19 pandemic. Potential participants were contacted through email or approached in-person with the study flier and invited to complete the study questionnaire. The in-person contact was added to the email contact to avoid bias of selecting only those with email addresses.

### Data collection

The COVID-19-Nutrition questionnaire, which is a researcher-developed pre-tested and validated questionnaire was used to obtain information on sociodemographic data, and changes in food purchase, consumption and handling pattern during the COVID-19 pandemic. The study questionnaire was developed by the investigators from literature [26,27]. The study assessment tools and procedures were pre-tested in a similar population, at a different location before the tools were implemented in the main study. Participants completed questionnaires in-person through face-to-face interviews. Participants were asked through 5-point Likert scales questions to answer whether their food purchase and consumption patterns were altered during the COVID-19 pandemic. The survey data collection occurred at a period when the quarantine restrictions has been lifted during January to March 2022.

### Statistical analysis

Chi-square tests and descriptive statistics were used to calculate frequencies, percentages, and to look for relationships. Changes in food purchasing, consumption and handling during the COVID-19 epidemic were the main output. The analysis did not include missing data. The Statistical Package for the Social Sciences (SPSS version 28.0) was used to analyze the data. Statistics were considered significant at P-values < 0.05.

## Results

There were 234 participants who completed the survey and 211 were included in the analysis for the primary outcome. Individuals who were approached and did not participate in the study was mainly because of lack of interest and time. Table 1 shows the sociodemographic characteristics of study participants and their association with type of household. Majority (43.6%) belonging to the 18–24 age range, and the female participants were more (62.1%). Approximately half (42.7%) of the participants were non-Hispanic Black. The majority of these participants completed high school (41.0%) as their highest level of education. Among the participants, 30.4% had full-time employment while 26.6% were full-time students and 115 (55.8%) had an annual income of less than $30,000. Nearly half (47.9%) lived in a SPH. There was a significant association between age, ethnicity, employment status, annual income and the type of household.

**Table 1:** Sociodemographic characteristics of study participants and their association with type of household (N = 211).

Variable	Single HH N = 101	Multiple HH N = 110	Total n (%)	P value
**Age (years)** 18–24 25–34 35–44 ^45^–54 55–64 65 and older	44 (43.6) 17 (16.8) 6 (5.9) 13 (12.9) 11 (10.9) 10 (10.0)	39 (35.5) 21 (19.1) 23 (20.9) 11 (10.0) 8 (7.3) 8 (7.3)	83 (39.3) 38 (18.0) 29 (13.7) 24 (11.4) 19 (9.0) 18 (8.5)	**0.030**
**Gender** Female Male	66 (65.3) 35 (34.7)	65 (59.1) 43 (39.1)	131 (62.1) 78 (37.0)	0.294
**Ethnicity** Non-Hispanic White Non-Hispanic Black Hispanic Asian Other	13 (12.9) 57 (56.4) 20 (19.8) 8 (7.9) 3 (3.0)	25 (22.7) 33 (36.7) 40 (36.4) 8 (7.3) 4 (3.6)	38 (18.0) 90 (42.7) 60 (28.4) 16 (7.6) 7 (3.3)	**0.002**
**Educational level** Less than high school Completed high school Completed two-year college Completed four-year college Completed graduate education Professional or doctorate degree	7 (7.0) 39 (39.0) 27 (27.0) 15 (15.0) 7 (7.0) 5 (5.0)	7 (6.4) 47 (42.7) 24 (21.8) 13 (11.8) 14 (12.7) 5 (4.5)	14 (6.7) 86 (41.0) 51 (24.3) 28 (13.3) 21 (10.0) 10 (4.8)	0.711
**Employment status** Full-time student Part-time employment Full-time employment Unemployed Retired	34 (34.3) 17 (17.2) 20 (20.2) 13 (13.1) 15 (15.2)	21 (19.4) 19 (17.6) 43 (39.8) 15 (13.8) 10 (9.3)	55 (26.6) 36 (17.4) 63 (30.4) 28 (13.6) 25 (11.8)	**0.020**
**Participation in Food assistance program** No SNAP/WIC benefits Received food from food pantry Received both SNAP benefits and food pantry	72 (72.0) 16 (16.0) 6 (6.0) 6 (6.0)	79 (71.8) 25 (22.7) 2 (1.8) 4 (3.6)	151 (71.9) 41 (19.5) 8 (3.8) 10 (4.8)	0.237
**Annual income** Less than $30,000	64 (64.3)	51 (29.2)	115 (55.8)	**0.015**

HH- household, Chi-square (*X*^2^), Fisher’s exact test, p<0.05.

**Fig 1:**
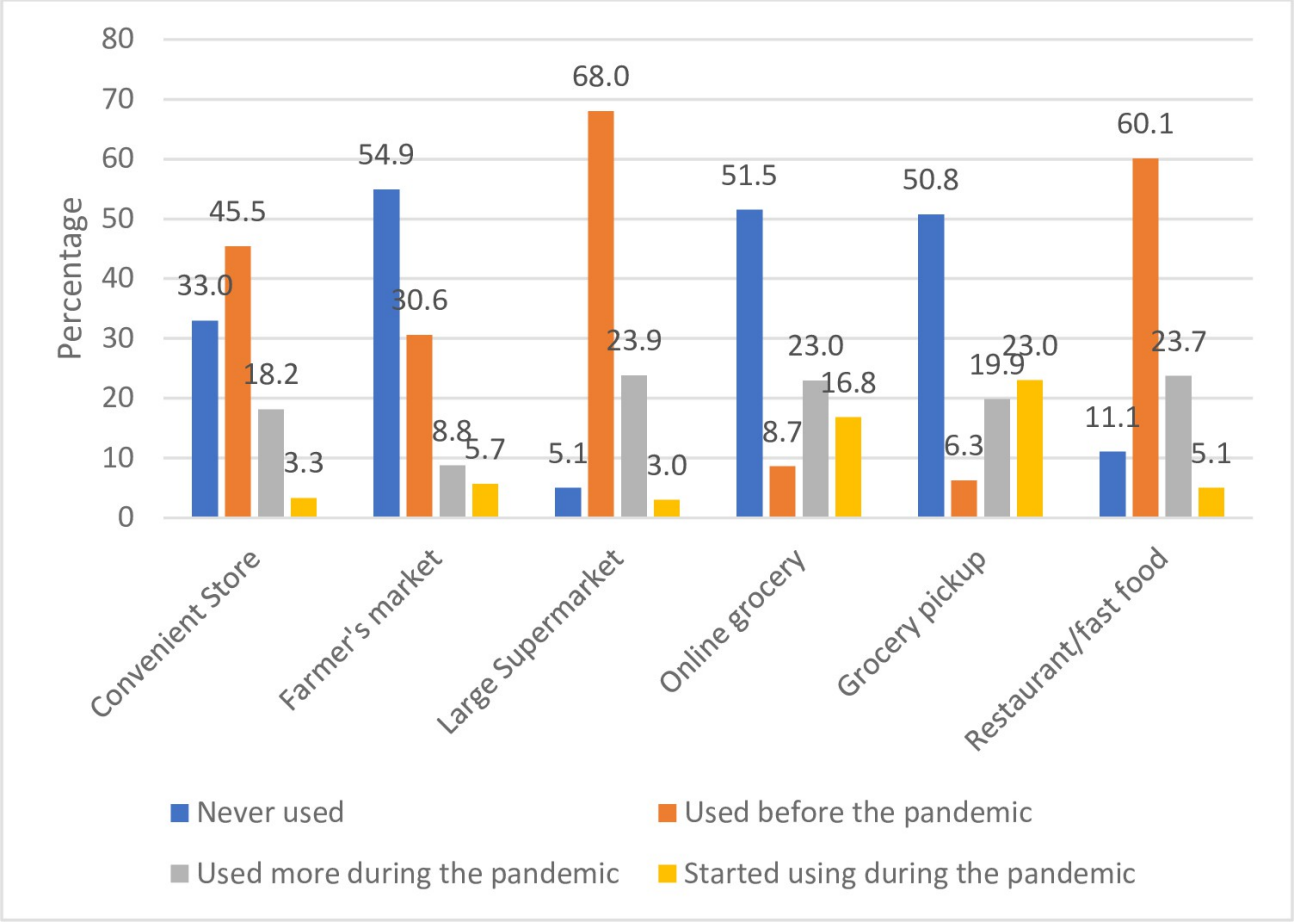
Food purchasing activities of participants during Covid-19.

Fig 1 displays the food purchasing activities of the participants during the COVID-19 pandemic. The largest proportion of participants never used farmers’ market (54.9%), online grocery (51.5% and grocery pickup services (50.8%) before the pandemic. Many of the participants used large supermarkets (68.0%), restaurant/fast food (60.1%), and convenient stores (45.5%) before the pandemic. Grocery pickup services (23.0%), and online grocery company (16.8%) were the highest purchasing source that participants started using during the pandemic.

**Fig 2:**
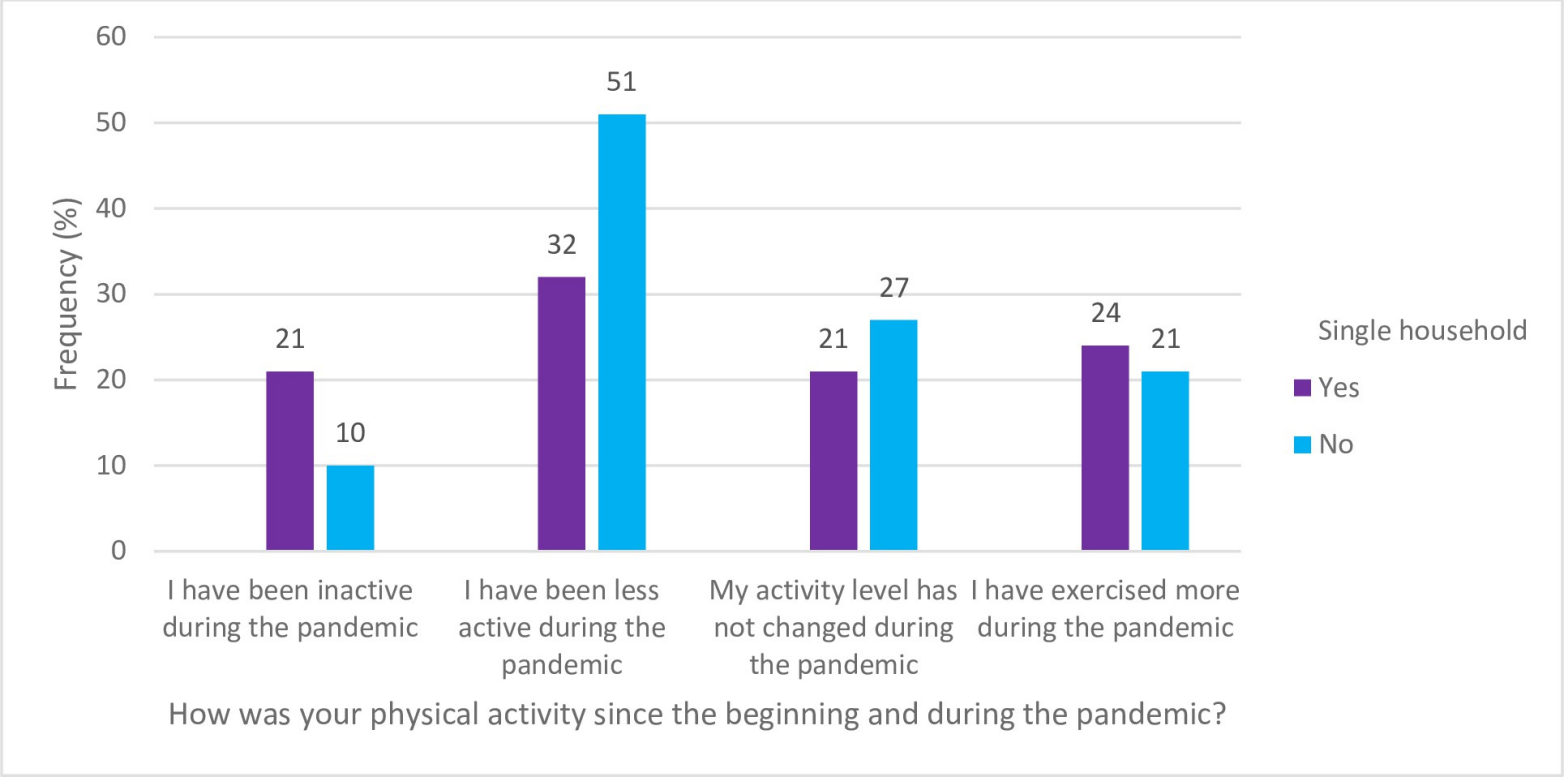
Physical activity of the participant during the COVID-19 pandemic.

**Fig 3:**
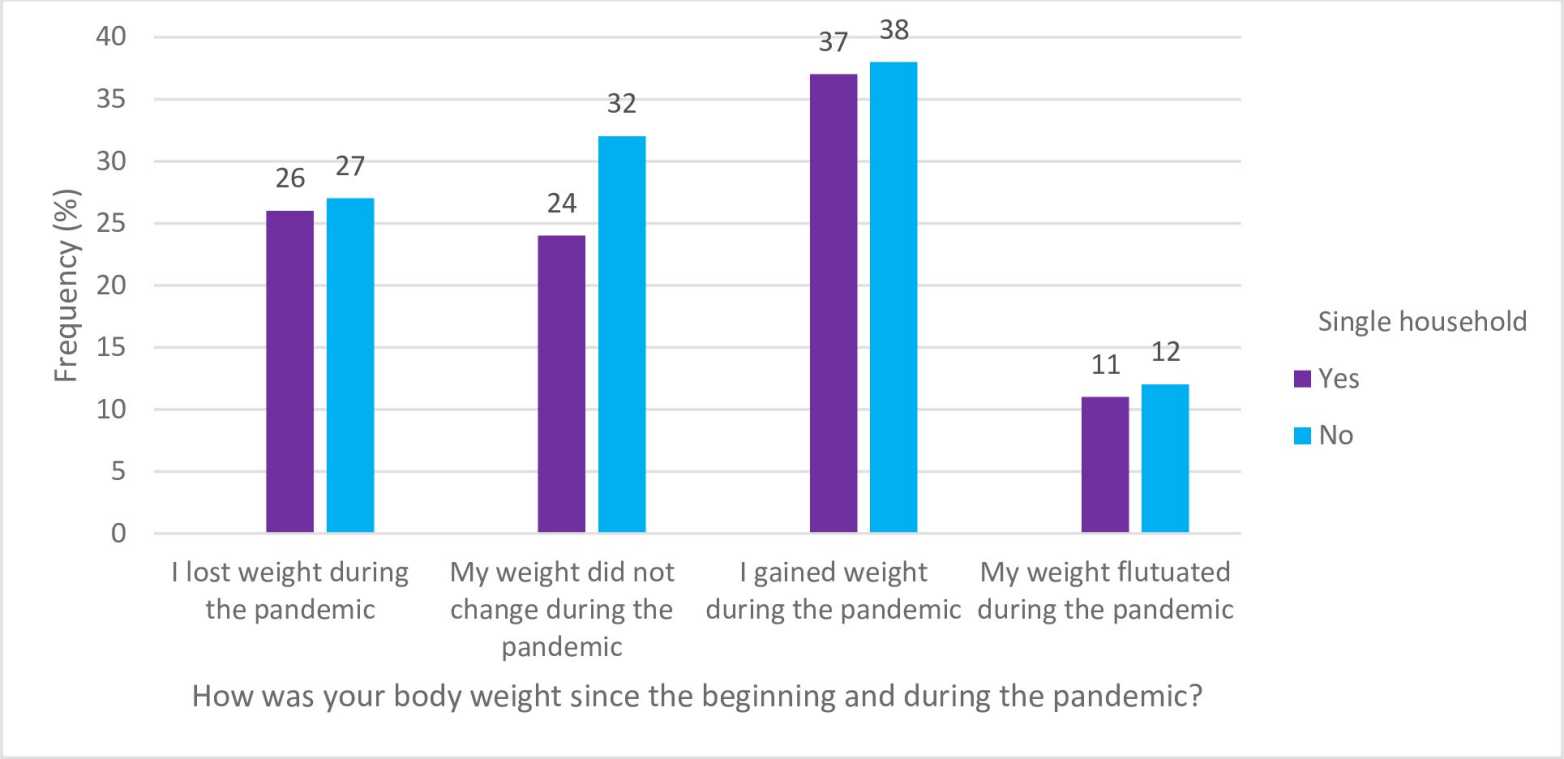
Changes in weight during the COVID-19 pandemic.

Fig 2 displays the physical activity of the participant during the COVID-19 pandemic. For the question, “How was your physical activity since before and during the pandemic?” the results are as follows; during the pandemic, 21% of SPH reported being inactive, 32% reported becoming less active, 24% exercised more during the pandemic while the activity level of 21% of SPH did not change. In answering the question, “How was your body weight since before and during the pandemic?” in Fig 3, about 37% of the SPH stated they gained weight during the pandemic and 11% of participants’ weight fluctuated during the pandemic.

Table 2 highlights the association between household type and food purchase and handling patterns during COVID-19. There was a statistically significant association between the household type and restaurant/ fast foods usage (*X*^2^ = 12.8, P = 0.002). The SPH had a higher proportion of those purchasing from the restaurant/ fast foods where 8% of SPH and only 2% of multiple person household reported that they started using the restaurant during the pandemic respectively. Similarly, 31% of SPH and 16% of multiple person households respectively reported using the restaurant/fastfood more during the pandemic. Table 3 shows the food consumption of the SPH before and during the pandemic. The larger proportion of consumption of all the food categories (meat– 57.0%, fish– 42.3%, tuna/salmon– 38.8%, bread/grain– 61.3%, salty snacks– 45.4%, frozen food– 48.5%, fruits– 55.7%, vegetables– 57.3%, eggs– 57.6%, milk/cereal– 43.8%, fast food– 36.7%, desserts [cookies, candy]– 39.8%, and ready-to-eat meals– 31.3%) stayed at the same amount of food before and during the COVID-19 pandemic. Conversely, 50.0% of study participants consumed more water, while 33.7% had less soda, and 44.8% had no alcohol intake before and during the pandemic. Table 4 shows the association of food consumption and handling behavior of the SPH with sociodemographic characteristics. There were significant associations between gender and the consumption of salty snack (P = 0.017) and deserts (P = 0.026), age group and consumption of meat (P = 0.003), ready-to-eat meals (P = 0.030), water intake (P = 0.031) and body weight (P = 0.020). Employment was associated with consumption of meat (P = 0.005), fast food consumption (P = 0.014) while the use of Federal Assistance program was associated with the consumption of bread/grain (P = 0.003), fruits (P = 0.003), Fast food (P = 0.045) and water (P = 0.001).

**Table 2:** Association of type of household with food purchase and handling pattern during the COVID-19.

Variable	Single person household	Multiple person household	*X* ^2^	*P*-value
**Mostly used food purchase source** Convenient store Farmers’ market Large supermarket Online grocery company Grocery pickup services Restaurants/fast foods **How was your cooking** Prepared less food Same as before Cooked more **How was your HH food wastage?** Wasted less food Same as before Wasted more	22 4 50 4 13 8 20 42 36 60 29 9	21 5 62 7 14 1 12 49 49 73 28 8	1.4 2.5 2.0 1.7 2.7 12.8 3.8 0.8	0.706 0.469 0.572 0.639 0.449 **0.002** 0.146 0.682

HH- household, Chi-square (*X*^2^), Fisher’s exact test, p<0.05.

**Table 3:** Food consumption of SPH before and during the COVID-19 pandemic.

Food/beverage item	Did not consume (%)	Consumed less (%)	Consumed same amount (%)	Consumed more (%)
Meat	9 (9.0)	21 (21.0)	**57 (57.0)**	13 (13)
Fish	18 (18.6)	26 (26.8)	**41 (42.3)**	12 (12.4)
Canned tuna/salmon	26 (26.5)	20 (20.4)	**38 (38.8)**	14 (14.3)
Bread/grain	2 (2.2)	15 (16.1)	**57 (61.3)**	19 (20.4)
Salty snacks	6 (6.2)	23 (23.)	**44 (45.4)**	24 (24.7)
Frozen food	6 (6.1)	18 (18.2)	**48 (48.5)**	27 (27.3)
Fruits	1 (1.0)	12 (12.4)	**54 (55.7)**	30 (30.9)
Vegetables	0 (0)	11 (11.5)	**55 (57.3)**	30 (31.3)
Eggs	3 (3.0)	13 (13.1)	**57 (57.6)**	26 (26.3)
Milk/cereal	10 (10.4)	21 (21.9)	**42 (43.8)**	23 (24.0)
Fast food	10 (10.2)	23 (23.5)	**36 (36.7)**	29 (29.6)
Desserts (cookies, candy)	8 (8.2)	27 (27.6)	**39 (39.8)**	24 (24.5)
Ready-to-eat meals	25 (25.3)	13 (13.1)	**31 (31.3)**	30 (30.3)
Water	2 (2.1)	8 (8.3)	38 (39.6)	**48 (50.0)**
Soda	19 (20.0)	**32 (33.7)**	30 (31.6)	14 (14.7)
Alcohol	**43 (44.8)**	19 (19.8)	16 (16.7)	18 (18.8)

**Table 4:** Association of food consumption and handling behavior of SPH with sociodemographic characteristics.

Food/beverage item	Gender (P-value)	Ethnicity	Age group(P-value)	Level of education (P-value)	Employment (P-value)	Use of Fed Assis Prog
Meat	0.669	0.183	**0.003**	0.211	**0.005**	0.163
Fish	0.868	0.314	0.251	0.529	0.164	0.167
Canned tuna/salmon	0.440	0.389	0.087	0.336	0.498	0.206
Bread/grain	0.456	0.268	0.725	0.992	0.238	**0.003**
Salty snacks	**0.017**	0.530	0.063	0.764	0.091	0.404
Frozen food	0.688	0.686	0.456	0.252	0.536	0.064
Fruits	0.294	0.197	0.132	0.766	0.950	**0.003**
Vegetables	0.268	0.218	0.220	0.781	0.920	0.234
Eggs	0.521	0.069	0.393	0.145	0.650	0.570
Milk/cereal	0.451	0.064	0.874	0.160	0.521	0.240
Fast food	0.897	0.579	0.071	0.163	**0.014**	**0.045**
Desserts	**0.026**	0.301	0.168	0.523	0.615	0.069
RTE meals	0.370	0.144	**0.030**	0.142	0.125	0.415
Water	0.916	0.599	**0.031**	0.135	0.603	**0.001**
Soda	0.380	0.138	0.163	0.478	0.191	0.785
Alcohol	0.799	0.192	0.228	0.659	0.696	0.334
Other variables						
Cooking	0.990	0.399	0.432	0.432	0.706	0.775
Food waste P. Activity Body weight	0.994 0.657 0.887	0.284 0.180 0.608	0.268 0.521 **0.020**	0.151 0.031 0.391	0.769 0.633 0.385	0.718 0.126 0.312

RTE- Ready-to-eat; P. Activity- Physical Activity.

## Discussion

In this study, the changes in food purchase, consumption and handling patterns were evaluated in relation to the COVID-19 pandemic with a focus on the single person households (SPH) as a unique household composition. Our study found that single person households (SPH) purchased more restaurant/fast foods. We found from assessment of the extent to which SPH changed their volume of food consumption, ranging from did not consume to consumed more, that across most food categories, food consumption remained the same for majority of participants, ranging between 35.7% and 58.2% of the sample. Aside from food consumption patterns, more people stated they consumed more water, less soda, and no alcohol during the COVID-19 pandemic.

Prior to the COVID-19 pandemic, a previous study [28] suggested that individuals living alone were more inclined to spend their money on dining out and restaurant services rather than on groceries. However, this article provides compelling evidence to demonstrate that the COVID-19 pandemic has brought about a substantial change in the spending habits of single person households. Our results are in line with the study conducted by Chenarides et al. [18] that observed that among their sample of urban dwellers, majority stated their food consumption patterns seemed to stay the same during the COVID-19 pandemic.

Our findings are contrary to other studies which recognized that the COVID-19 pandemic has had a significant impact on food purchasing behaviors, with many individuals moving away from restaurant meals and increasing their visits to grocery stores [17,29,30]. However, this study observed that the SPH had a higher proportion of those purchasing from the restaurant/ fast foods. The reason for this could be attributed to the timing of data collection in 2021, which coincided with the lifting of the lockdown and easing of COVID-19 measures. At this point, there were no more requirements for masks or social distancing, and restaurants were operating at full capacity as opposed to being shut down during the peak of the pandemic.

In our study, majority (71.9%) of the participants did not participate in food assistance programs and there was no significant association between type of household and participation in food assistance program. This is contrary to previous studies in the US [31–34] that observed a peak in visits to food banks and pantries and farmers’ markets in December of 2020 because a number of households experienced food hardship and insecurity amid the COVID-19 pandemic. The use of Federal assistance program was however associated with consumption of fruits and bread/grain among the SPH. This could be attributed to the fact that COVID-19 pandemic impacted the availability and affordability of fruits and vegetables [35] and compared to multiple household participants, majority of SPH were low income earners, Blacks and either full time students or retired.

Among the entire study population, our assessment of whether the participants utilized in-store grocery shopping and online grocery shopping, ranging from never used to increased use of grocery shopping, showed that many participants started using grocery pickup and online grocery shopping during the pandemic. The findings of our study support previous research indicating a rise in online grocery shopping and grocery pickup services during the COVID-19 pandemic [36,37]. Our study also found that the pandemic has led to increased acceptance of grocery pickup among the participants in agreement with previous findings [17,38]. Interestingly, a substantial number of our respondents had no prior experience with online grocery shopping and grocery pickup services. This differs from previous studies reporting an upward trend in these services [19,32,38]. Our study participants were mostly individuals ranging from ages 18–24 years. Younger ages between 18–34 years have been seen to be more open to the idea of online grocery shopping [32]. This could account for the peak in online grocery shopping and the utilization of grocery pickup services. However, this age group was most likely to engage in indoor dining and restaurant purchases during a time where the pandemic was the most dangerous, this period covers December 2020 to March 2021. Another study [26] found that ages 19–26 years had a higher percentage of ordering take out. The increasing demand for grocery delivery and pickup services presents an opportunity for retailers to capitalize on this trend. Retailers with strong logistical capabilities are better positioned to incorporate grocery delivery as a permanent service, as these modes of shopping are expected to gain further traction among consumers in the future [39]. Retailers can do so by implementing programs that make it easy for customers to access these services.

Additional findings in our general study population were in relation to physical activity and body weight. More individuals noted decreased physical activity and weight gain throughout the pandemic. Investigators looked into how the COVID-19 pandemic affected people’s levels of physical activity and weight gain. Our findings support previous research indicating that the pandemic has led to significant weight gain among many individuals [40,41]. This may be as a result of the pandemic’s stress and anxiety contributing to unhealthy eating habits [42,43]. Moreover, the isolation resulted in remote work options, leading to decreased physical activity and further exacerbating weight gain [44]. The increased availability of food delivery options from restaurants during the pandemic was found to have contributed to reliance on fast food, further contributing to weight gain [42]. This highlights the need for individuals to adopt healthy eating habits and limit fast food consumption during the pandemic. Moreover, individuals should seek alternative ways of coping with stress and anxiety that do not involve unhealthy food choices [31,42,44,45]. Physical activity should be promoted, particularly among those living alone, as it may positively impact weight management and overall health outcomes. Many turned to online grocery shopping as a way to reduce their risk of exposure to the virus. The impact of the COVID-19 pandemic on weight gain and physical activity may have been influenced by several demographic factors including ethnicity, education level, income, household size, employment status and may have varied based on individual circumstances such as access to social support networks.

Across most food categories, food consumption remained the same for majority of participants before and during the COVID-19 pandemic. This may be connected to the income level among the SPH participants, more than half (64.3%) had an annual income below $30,000. This low income may have affected their purchasing power to make changes to their food consumption behavior, and therefore stayed same. Our study suggested that employment status was significantly associated with meat consumption. This implies that individuals’ work situations may influence their tendency to consume meat. In a study that assessed COVID-19 impact on U.S. households, it was found that even among middle-class households (income < $50,000, or between $50,000 and $99,999), it was less likely to observe increase in their grocery expenditure during the pandemic [33]. In addition, we recognized that there was an increase in water intake and a decrease in soda consumption, our results showed that 44.8% of the sample size did not consume alcohol since the COVID-19 pandemic. Naicker et al. [35] also found a significant increase in water consumption during the COVID-19 pandemic in the sample studied, and this finding was similarly reported by Kent et al. [25].

There are some limitations to our study that should be acknowledged. First, our sample size was not so large and were from two counties of Texas, which may limit the generalizability of our findings to other single person household types. Second, our data was based on self-reported expenditures and consumption, which may be subject to recall bias and social desirability bias. Third, our study only examined the short-term impacts of the pandemic on food purchase, consumption and handling. Further research is needed to investigate longer-term trends and changes in dietary habits, also the impact that health conditions, dietary preferences may have on these patterns. In addition, the period of data collection which is after the lockdown may have affected the study outcome.

## Conclusion

This study provides insights into the consumption of restaurant/fast foods, and food handling among single person households during the COVID-19 pandemic. Our findings suggest that single person households spent a significant portion of their income on restaurant/fast food. Our study also adds to the growing body of research highlighting the importance of online grocery shopping and grocery pickup services during the COVID-19 pandemic. The results from the study have the potential to assist public policy officials, food service, and health authorities predict how different categories of consumers will react in pandemics and to develop interventions to promote healthy eating habits during such challenging times. It may also be used to inform area-specific alleviation strategies to minimize the impact of the COVID-19 pandemic and future events especially in this age when single-person household consumer’s lifestyle, products and services being developed and offered by the food service industry have been forced to adapt to change [10].

## Supporting information

S1 File COVID-19 pandemic-nutrition dataset. (XLSX)
